# The prevalence of alcohol-involved crashes across high and low complexity road environments: Does knowing where drinking drivers crash help explain why they crash?

**DOI:** 10.1371/journal.pone.0266459

**Published:** 2022-04-20

**Authors:** Mark B. Johnson

**Affiliations:** National Capital Region Center, Pacific Institute for Research and Evaluation, Beltsville, Maryland, United States of America; Tsinghua University, CHINA

## Abstract

**Objective:**

Alcohol use has been linked to impairment of cognitive and psychomotor driving skills, yet the extent to which skill impairment contributes to actual crashes is unknown. A reasonable assumption is that some driving situations have higher skill demands than others. We contend that intersections, the presence of other vehicles or moving objects, and work zones are examples of common situations with higher skill demands. Accordingly, if skill deficits are largely responsible for alcohol-involved crashes, crashes involving a drinking driver (versus only sober drivers) should be overrepresented in these driving situations.

**Method:**

Publicly available FARS data from 2010 to 2017 were collected. Fatal crashes were coded as alcohol-involved (1+ driver with a blood alcohol concentration [BAC] ≥ .05 g/dl) or having no impaired driver (BACs = .000). Drug-positive crashes were excluded. Crashes were also coded as involving moving versus stationary objects, occurring *at* versus *away* from intersections, being multivehicle versus single vehicle, occurring *at* or *away* from work zones.

**Results:**

Across multiple models, controlling for time of day and type of road, alcohol-involved crashes were significantly *underrepresented* in crashes at intersections, with moving objects, and other vehicles. Most strikingly, alcohol-involved crashes were 24 percentage points more likely to be with a stationary object than a moving object.

**Conclusions:**

No evidence supported the idea that skill reductions are a primary contributor to alcohol-involved crashes. Alternative explanations and limitations are discussed.

## Introduction

Across numerous studies, alcohol consumption has been reliably related to motor vehicle crashes in a dose-dependent fashion; higher blood alcohol concentrations (BACs) are associated with exponentially increasing risk of crash involvement [1–6]. Dovetailing with this epidemiological evidence are experimental studies showing alcohol-induced impairment on a wide variety of cognitive and psychomotor skills ostensibly related to safe driving [7, 8].

The ubiquitousness of controlled dosing studies on driving-related skills in the published literature underscores the widespread belief that alcohol consumption increases crash risk *because* it diminishes these driving skills. Alcohol affects driving skills, for example, by impairing drivers’ reaction times, motor coordination, visual tracking, judgment, divided attention, and so forth [7, 8]. And it is not difficult to imagine how drinking drivers might crash if their reactions were slowed, coordination was hampered, attention was limited, etc.

However, there is little understanding about *how* skill impairment translates into actual crashes; we don’t know the extent to which quantitative decrements in these individual skills (or combinations thereof) are represented as causes of real crashes in the population. It is logical to assume, for example, that the population-level impact of impaired *reaction time* depends, in part, on the prevalence of driving situations that require quick reactions. Further, it assumes driving situations where the difference in reaction speed between a sober and alcohol-impaired driver is enough to avoid the collision. This reasoning applies to the various other driving skills, such as coordination and divided attention, examined in the literature.

The population prevalence of these specific driving situations—events where alcohol impairment of various driving skills is exposed—is unknown. But we can reasonably assert that some driving situations demand higher levels of driving skills than others. For example, driving through intersections should require greater attention, coordination, etc., than driving on roads away from intersections or exchanges. Similarly, driving in proximity to other vehicles/moving objects, or through work zones, should require greater skill functioning than situations where there are no other vehicles, away from work zones, etc. And while it is difficult to articulate which specific skills are needed under each specific environment, previous research has defined, or has experimentally constructed, roadways with intersections, junctions, and heavier traffic/other moving objects as ‘high complexity’ and requiring greater skill and attentional resources [9–11].

In a thought experiment, we might consider two driving situations: (A) a road with no other vehicles, no intersections or exchanges to navigate, no other moving objects to deal with, etc., and thus lower skill demands, and (B) a road at an intersection or exchange with other vehicles or moving objects, and thus higher skill demands. If the *general setting* (e.g., type of road, urban versus rural, time of day or night, etc.) is held constant to equate exposure, what might we expect in terms of alcohol-involved versus sober crashes? It is easy to imagine that in situation A, where skill demands are lower, the difference in crash rate between a sober and alcohol-impaired driver might be small, but the crash rate would be exacerbated under situation B. In the latter, where the demand for driving skills is higher, we would expect reduced skill levels due to alcohol to manifest as increased risk for crash involvement. Accordingly, to the extent alcohol consumption contributes to crashes by impairing important driving skills, we would expect alcohol-involved crashes to occur at a higher rate in situations where more skillful driving and more focused attention are required [12, 13].

In this report we carry out the previously described thought experiment using data from the Fatality Analysis Reporting System (FARS). We examined the rates of sober versus alcohol-involved fatal crashes under four interrelated driving situations: whether the vehicle crash was with a moving versus stationary object, whether the crash occurred at an intersection, whether the crash occurred at construction zones, and whether the collision involved single versus multiple vehicles. Evidence that alcohol-involved crashes are overrepresented in situations with higher skill demands would help bridge the gap between experimental and epidemiological research on impaired driving. It would support the idea that the experimental research on alcohol and impairment of driving skills is applicable to understanding real-world crashes.

## Materials and methods

### Data used

This study analyzed 2010 to 2017 FARS data. Data can be downloaded at https://www.nhtsa.gov/file-downloads?p=nhtsa/downloads/FARS/. ACCIDENT files for the corresponding years were downloaded and combined, producing a total of 253,273 crashes. From the Vehicle Total variable, we determined whether a crash was single vehicle (n = 145,033) or involved more than one vehicle (n = 108,420). From the Type of Intersection, we computed whether the crash occurred at any of seven different types of intersections listed in the FARS coding manual (n = 59,809) or whether it was not at an intersection (n = 193,091). Similarly, from the Work Zone variable, we computed whether the accident occurred at any of four different types of work zones listed in the FARS coding manual (n = 4,816) or whether it was not in a work zone (n = 248,404). Finally, from the First Harmful Event variable, we computed whether the accident was with a moving object (n = 140,166) or stationary object (n = 74,797). Moving objects included pedestrians, pedacyclists, railway vehicles, live animals, and of course other motor vehicles in transit. Stationary objects included parked vehicles, buildings, poles, barriers, trees, and other construction pieces (codes 19 to 43 in the FARS coding manual).

We also recoded time of crash into six four-hour blocks, beginning with 5 a.m. to 8:59 a.m. Further, the ROAD_FNC (2010–2014) and FUNC_SYS variables (2015–2017) were used to indicate type of road (interstate, expressway, principal arterial, minor arterial, collector, local, and unknown). Similarly, ROAD_FNC (2010–2014) and RUR_URB (2015–2107) were used to compute a land use variable (rural, urban, or unknown). ROAD_FNC identified roads both by road type and rural versus urban, while FUNC_SYS and RUR_URB simply coded roads by type, and by rural versus urban, respectively. These were used as control variables in all analyses.

The accident data were supplemented by information from the PERSON files in FARS, which contained drinking and drug use information. Each crash was identified as involving: (a) at least one driver with a BAC at or above .05 g/dl (22.98%), (b) at least one driver with a BAC between .000 g/dl and .049 g/dl (2.4%, not analyzed), (c) all drivers with a BAC of .000 g/dl (38.27%), or (d) drivers not tested for alcohol (36.35%, not analyzed). To control for drug use, we also coded crashes as involving a (a) drug-positive driver (20.30%, not analyzed), (b) drug-negative driver (18.10%), or (c) driver not tested for drugs (61.60%). The decision to test or not test drivers for drugs is made by local law enforcement and forensic laboratories as part of standard data collection for FARS.

### Analytic approach

We used the generalized linear model with a logit link function (logistic regression) to predict whether the crash occurred in a high skill demand situation versus less skill demand situation as a function of whether the crash was alcohol-involved. The *generalized linear model* is a broad analytic approach for analyzing data with outcomes that fall in any number of distributional categories within a single framework [14]. For binary outcomes, the logit of outcome probability (*θ*) (see below) is used as the response variable in the regression, which then follows the rules of normal regression. This approach allows the analysis to draw on the F-distribution, which is superior to non-parametric approaches when it comes to Type I error control while preserving power and accuracy of estimation [15, 16].

Specifically, we used alcohol involvement to predict whether crashes involved a moving versus stationary object, took place at or away from an intersection, took place at or away from a work zone, or involved a single vehicle or multiple vehicles. In all cases we controlled for time block, road type, and land use. The formal model is presented below.


$$\mathrm{Logit}\;(\theta )=\ln\;(\frac{\theta }{1-\theta })=\beta_{0}+\beta_{1}\mathrm{Alc}+\beta_{2}\mathrm{Time}+\beta_{3}\;\mathrm{RoadType}+\beta_{4}\;\mathrm{LandUse}$$

Where *θ* is the probability of a crash (moving versus stationary object, intersection versus non-intersection, work zone versus non-work zone, and multi-vehicle versus single vehicle), β_0_ is in the intercept, β_1_ is the predictor of interest (alcohol-involved versus sober), and β_2–4_ reflect the covariates.

## Results

### Main analysis

We tested three models for each of the four outcomes, primarily to address limitations of FARS data [17]. For each outcome, our primary model (Model 1) compared crashes where a driver had a BAC ≥ .05 g/dl versus crashes where all drivers had tested negative for alcohol. Crashes where a driver tested positive for drugs were excluded, but crashes where drivers weren’t even tested were included in the analysis. Model 2 was identical to Model 1 except analysis was limited to nighttime hours (two time-blocks, 9 p.m. to 4:59 a.m.), thus controlling for time methodologically rather than statistically. Finally, Model 3 was like Model 1 except only crashes where drivers tested negative for drugs were included (crashes lacking drug tests were excluded). We did not anticipate meaningful differences among the three models but included them to rule out missing data in FARS, or time of day, as an explanation for results.

Results are summarized in Table 1. Under all three models, alcohol status predicted whether crashes were with stationary or moving objects, away from intersections or with intersections, and involved single or multiple vehicles. Alcohol status did not significantly predict whether crashes occurred away from a work zone. Road type, land use, and time block were significant predictors in each analysis (*p* < .001), but we do not discuss or interpret them in this brief report.

**Table 1:** Adjusted Odds ratios of alcohol-involved drivers crashing in relatively more complex environments under different analytic assumptions.

Effects	Moving Object	Intersection	Work Zone	Multi Vehicle
*Model 1*				
BAC ≥ .05 g/dl	0.35 (0.34–0.36) *	0.60 (0.58–0.63) *	1.01 (0.91–1.12) ^NS^	0.63 (0.61–0.65) *
*Model 2*				
BAC ≥ .05 g/dl	0.37 (0.35–0.39)* *	0.76 (0.72–0.81) *	1.06 (0.91–1.23) ^NS^	0.82 (0.79–0.86) *
*Model 3*				
BAC ≥ .05 g/dl	0.21 (0.20–0.22)*	0.61 (0.57–0.65) *	1.03 (0.86–1.23) ^NS^	0.64 (0.60–0.68) *

* *p* < .0001.

In the results below, odds ratios are coded to reflect situations with higher skill demand. If alcohol contributes to increased risk in settings with greater skill demands, we would expect larger odds ratios for alcohol-involved crashes, indicating that alcohol-involved crashes are overrepresented in those settings (i.e., collisions with a moving object, at an intersection, at a work zone, and involving other vehicles).

In no case were alcohol-involved crashes significantly more likely to occur in settings with higher skill demand. To the contrary, in every situation except work zones, alcohol-involved crashes were significantly *underrepresented* in situations with higher skill demands; crashes with a drinking driver were *less* likely to occur with moving versus stationary objects, at intersections versus away from intersections, and involving multiple versus single vehicles. This is true even when analysis was restricted to nighttime hours (Model 2), and when the sample was reduced to crashes where drivers definitively tested negative for drugs (Model 3). The analyses also controlled for type of road and whether the setting for urban or rural to help equate exposure.

Note that relatively few crashes in general (1.9%) occurred in work zones, and when broken down by alcohol-involvement and seven different types of road, counts in some cells were quite small. However, when road type was removed as a control variable, alcohol status significantly predicted work zone crashes in the same direction as the other situations (in all three models, *p*-values < .05, adjusted OR ~ 0.83).

For a different perspective of the size of the effects, we provide fitted proportions for sober versus alcohol-involved crashes for each of three situations (Model 1): crashes with moving objects (73.6% of sober crashes versus 49.3% of alcohol-involved crashes), crashes involving multiple vehicles (58.5% versus 47.0%), and crashes at intersections (17.4% versus 11.4%).

### Exploratory analysis

We decided *post hoc* to examine sober and alcohol-involved crash rates as a function of type of road. Crashes occurring on interstates and freeways/expressways were combined into a “high speed road” category (n = 41,227), while crashes occurring on principal arterial, minor arterial, collector, and local roads were combined into a “lower speed road” category (n = 210,199). Crashes on roads of unknown category were excluded. We modeled Alcohol Crash Status × Road Category interactions for collisions with moving objects and multivehicle collisions only, given the very small counts of work zone crashes in general and the fact that interstates and freeways should include very few intersections.

For collisions with moving objects, the interaction was statistically significant, *F*(1, 96253) = 750.6, *p* < .0001. Alcohol-involved crashes were underrepresented in crashes with moving objects (e.g., moving objects) to a smaller extent on faster roadways (62.5% vs. 56.8% for sober vs. alcohol-involved crashes) relative to arterial and slower roadways (76.1% vs. 46.3%). The interaction also was significant regarding multivehicle collisions, *F*(1, 112E3) = 541.8, *p* < .0001. On lower speed roadways, alcohol-involved crashes were underrepresented in multicar collision situations (64.0% vs. 46.2%). But on faster roadways alcohol-involved crashes were slightly overrepresented (61.7% vs. 64.4%). All stated comparisons were statistically significant (all *p*-values < .0005).

### Sensitivity analysis

It is possible that our observed results are due to bias in how police officers or forensic laboratories chose to test all drivers for alcohol. For example, police officers might have missed some alcohol-involved crashes by not testing all drivers, thus affecting the results. Table 2 shows raw counts of crashes that fall into the BAC and moving object versus stationary object categories used the analysis. This table ignores/excludes crashes where the highest measured BAC was greater than .000 g/dl but lower than .05 g/dl, but we provide counts for crashes where drivers were not tested for alcohol.

**Table 2:** Raw counts of crashes for stationary and moving object crashes as a function of alcohol crash status.

	Not Tested	All BACs = .00	High BAC ≥ .05	Total
**Stationary Object**	20562	24691	27370	*72623*
**Moving Object**	58956	58869	18948	*136773*
**Total**	*79518*	*83560*	*46318*	*209396*

Examining crashes as a function of involvement with moving versus stationary objects, for example, we see that in 28.3% (20,562 / 72,623) of crashes with stationary objects, the driver(s) were not tested for alcohol, but 43.1% (58,956 / 136,773) of crashes with moving objects involved no alcohol testing. Further, based on these raw data (not model adjusted), 70.4% (58,869 / 83,560) of sober crashes involved moving objects, whereas only 40.9% (18,948 / 46,318) of alcohol-involved crashes involved moving objects.

One can argue that decisions to test or not test crash-involved drivers were driven by ‘facts on the ground’ and generally accurate, and the decisions to not engage in testing were based on time and cost factors. If all non-tested cases were treated as sober crashes, then the percentage of sober crashes that involved moving objects would move to 72.2%, which is not meaningfully different from 70.4% and does not change the interpretation of the findings.

But what if some of the non-tested moving object crashes involved a drinking driver and were missed by police? A difference of 14.8% (43.1%– 28.3%) and a total of 20,231 (14.8% x 136,773) *more* moving object crashes than stationary object crashes did not test all drivers. What if all of the non-tested stationary object crashes (28.3% of all stationary object crashes) in fact were sober, and a comparable 28.3% of total moving object crashes were sober, but the excess 20,231 non-tested moving object crashes (14.8%) were actually alcohol-involved? Even then, alcohol-involved crashes would be underrepresented in moving object crashes (58.9%, (20,231 + 18,948) / (20,231 + 46,318) relative to sober crashes (68.3%, ([58,956 –20,231] + 58,869) / ([58, 956 – 20,231] + 58,869 + 45,253). Recall the expectation presented in the introduction suggested that alcohol-involved crashes would be overrepresented in crashes with moving objects, other vehicles, and at intersections.

## Discussion

We argue that safely navigating driving situations with intersections or with other moving objects or vehicles has higher driving skill requirements than situations away from intersections and without moving objects or other vehicles [9–11]. Because alcohol has been shown experimentally to impair these driving skills, it is reasonable to expect alcohol-involved crashes to be overrepresented in crashes at intersections, and with moving objects or other vehicles. Evidence of such would support that idea that experimental driving skill measures are important in explaining real-world crashes.

However, our examination of FARS data found the opposite. For most tests, alcohol-impaired fatal crashes were overrepresented in situations with lower skill demands. For example, over 75% of sober fatal crashes involved another moving object, whereas slightly less than half of alcohol-involved crashes involved another moving object. These results, at face value, generally are not consistent with the idea that impairment of driving-related skills explains alcohol-involved crashes. While one limitation of the research is the lack of individual difference measures (e.g., demographics, personality, driving style) in our *crash-level* analysis, it does not seem highly likely that these factors would be distributed unevenly among environments. For example, it is unlikely that risk-taking male drivers avoid intersections by such a wide margin at the population level.

Had the paper presented counterfactual findings, it is unlikely they would be considered controversial. Arguing first that alcohol impairs attention, reaction time, motor coordination, etc., and then finding that drinking drivers tend to be involved in serious crashes in situations with higher demands for attention, reaction time, motor coordination, etc., would not be surprising. However, statistically significant and meaningful patterns in the other direction suggest a potential disconnect between our understanding of driving risk as informed by the experimental literature and what happens in real crashes. It is possible, at least, that the relationship between driving skills and real crash risk is not clear as once thought.

First, however, we need to consider the possibility that results are artifactual and due to features of the design. Importantly, our analytic model did attempt to control for exposure to the extent the relevant data items were available to do so. We controlled for time of night, type of road, and rural versus urban for over 200,000 crashes. We also used different approaches to exclude drug positive driving, and the results were relatively persistent. Given these controls, it seems unlikely that the results would be caused by drinking drivers disproportionately traveling on roads without intersections or other vehicles. In fact, people tend to live, and commercial establishments are located, where other people tend to be. Differences in exposure to intersections, moving objects, etc., does not appear to be a viable explanation for the size of the differences observed.

We also note the possibility that there is bias in the FARS data regarding when crash drivers are tested for alcohol. Perhaps our observed results reflect the fact that crashes where drivers are never tested for alcohol in fact include alcohol-involved crashes, but that these are distributed unevenly between moving and stationary object crashes? However, in our sensitivity analysis we demonstrate that even under assumptions that a large number of non-tested moving object fatal crashes are actually alcohol-involved does not undo the most striking observed results.

Of course, FARS involves only fatal crashes. We are taking the conservative approach and generalizing findings only to crashes that involved a fatality. Still, our analyses found that alcohol-involved crashes are overrepresented in simpler driving situations primarily on moderate to slower speed roads, and less so on interstates and highway. Given that vehicle speed is a primary factor in crash fatalities and that lower speeds are less likely to be fatal, it is reasonable that the results would apply as well to non-fatal crashes. However, we do not have direct evidence showing this relationship

So, what does it mean that fatal crashes involving alcohol-impaired drivers don’t appear well represented in situations where we think they should be? Either alcohol-involved drivers (BAC ≥ .05 g/dl) don’t suffer from skill impairment, navigating through intersections and/or dealing with other vehicles don’t require higher levels of skill functioning, or the driving skills in question aren’t directly instrumental in fatal crashes on a large scale. It is worth noting that in Michaels et al.’s driving simulator experiment [9], individual differences in perceptual-cognitive skills (3D object tracking) significantly predicted crashes *only* in moderately complex rural settings but not in more complex urban settings.

We argue the latter is most plausible. It is easy to imagine how delays in reaction time, or reduced attentional capacity, or poor coordination *could* lead to crashes, but these really require specific circumstances within intersections, encounters with moving objects, etc.—circumstances where the quantitative decrements skill performance induced by alcohol will make a difference between involvement in a fatal crash or not. Yet, it is unclear how frequent these circumstances are on the population level.

If not due to decrements in skill performance, then what explains the pattern of results? It is unlikely there is one single explanation, but we have argued that the sedating effects of alcohol and drugs (as opposed to the *intoxicating* effects) are an understudied contributor of impaired driving crashes [18–20]. While drowsiness produces quantitative decrements in driving-related skills similar to alcohol and some drugs [21], we argue that falling asleep behind the wheel (for which drowsiness is a precursor) represents a *qualitative* change in crash risk. Crashes due to quantitative skill impairment require there being objects to track and avoid, things to drive around and react to, situations requiring decision making, etc. Falling asleep behind the wheel, however, can lead to a crash even in the most barren driving environments.

While things like other vehicles and traffic lights may be risk factors for drivers struggling with attentional and psychomotor deficits because they add visual stimulation, it is possible they are protective against falling asleep behind the wheel. For example, monotonous driving environments such as long rural highways contribute to “highway hypnosis” [22], but the introduction of novel stimuli can limit this effect. Along these lines, it is not difficult to imagine that encountering new events on the road—an intersection or even another vehicle—might help prevent drowsy drivers (whether induced by alcohol or sleep deprivation) from falling entirely into somnolence. They also may cue drinking drivers to try to compensate for their impairment [23]. The fact that the difference in rates of sober versus alcohol-involved crashes were greatest on moderate and slower roads (e.g., arterial roadways), where these visual cues or stimuli are more likely to be prevalent, is consistent with this idea.

It is further worth noting that alcohol use has been associated with increased aggression [24–27], and some alcohol-involved crashes may result from aggressive driving and speeding. While this explanation is quite than our hypothesis of alcohol-induced sedation, it still reflects plausible examples of crash causality that does not rely on impairment of driving related skills. And lack of roadway complexity (e.g., few intersections or other vehicles) may facilitate aggressive driving behavior such as speeding.

We have no *direct* evidence to show that attentional and cognitive driving skills are minimally important in understanding drinking and driving risk, nor that sedation and aggression (as opposed to skill impairment) are primary causes of impaired driving fatal crashes, nor that the presence of traffic lights and other vehicles can be protective against falling asleep behind the wheel. But all three are consistent, at least, with the data presented. Admittedly, our analysis of FARS data was simplistic, and our selection of ‘high skill demand’ environments (e.g., intersections, moving objects/other vehicles and work zones) was limited. Perhaps these situations are too broad and insensitive to the sort of variability in driving skills we see among drinking drivers. But, as a whole, these situations are quite common, and to the extent that alcohol-induced skill impairment requires more specific circumstances to manifest as increased risk of fatal crashes, the less relevant they must be in terms of explaining public harm.

In the end, the research presented herein raises more questions than it answers. And that is the point. The analytic results of a hundred thousand or so crashes do not fit with the common understanding of how alcohol contributes to fatal crashes, and this is deserving of discussion.

One consequence of this research is that it underscores the need for more work on the sedating effects of alcohol as a crash risk factor. Robust and replicated evidence that alcohol-involved drivers are disproportionately likely to fall asleep at the wheel has policy and public health implications. It could, for example, serve as impetus for establishing a lower late-night BAC *per se* limit. Restricting a lower BAC limit to late-night hours/after midnight (when drivers are naturally most drowsy) might be politically acceptable.

A second, less direct consequence of our work is that researchers may more carefully consider the utility of standard laboratory studies on driving impairment. Without doubt, experimental, double-blind, placebo-controlled methods are invaluable for understanding the causal effects of alcohol and drugs. Advancements in epidemiological research, however, could improve the generalizability and ecological validity of laboratory results. For example, identifying which skill deficits appear central to real crashes could inform how to design laboratory experiments. Or data from the naturalistic driving studies (e.g., https://insight.shrp2nds.us/) might be used to develop driving simulator scenarios that are definitively representative of actual crashes.

Both have implications for greater understanding of the etiology and prevention of impaired driving crashes and deserve additional scientific attention. Future research needs to include data on individual differences among drivers (e.g., demographics, personality, driving style and exposure) as well as more environment features such as enforcement, specific traffic conditions, and weather. These are critical to better understanding the role of driver skill in alcohol-involved driving.
